# Morphological and Behavioral Convergence in Extinct and Extant Bugs: The Systematics and Biology of a New Unusual Fossil Lace Bug from the Eocene

**DOI:** 10.1371/journal.pone.0133330

**Published:** 2015-08-12

**Authors:** Torsten Wappler, Eric Guilbert, Conrad C. Labandeira, Thomas Hörnschemeyer, Sonja Wedmann

**Affiliations:** 1 Senckenberg Forschungsinstitut und Naturmuseum Frankfurt, Forschungsstation Grube Messel, Messel, Germany; 2 Senckenberg Biodiversität und Klima Forschungszentrum (BiK-F), Frankfurt am Main, Germany; 3 Steinmann-Institut für Geologie, Mineralogie, Paläontologie, Universität Bonn, Bonn, Germany; 4 Muséum National d’Histoire Naturelle, Département de Systématique et Evolution, UMR 7205 CNRS, CP50, Paris, France; 5 Department of Paleobiology, National Museum of Natural History, Smithsonian Institution, Washington, DC, United States of America; 6 Department of Entomology and Behavior, Ecology, Evolution and Systematics Program, University of Maryland, College Park, MD, United States of America; 7 College of Life Sciences, Capital Normal University, Beijing, China; 8 Georg-August-Universität Göttingen, Johann-Friedrich-Blumenbach-Institut für Zoologie & Anthropologie, Abteilung Morphologie & Systematik mit Zoologischem Museum, Göttingen, Germany; UMR INRA/INSA, BF2I, FRANCE

## Abstract

The bug *Gyaclavator kohlsi* Wappler, Guilbert, Wedmann et Labandeira, gen. et sp. nov., represents a new extinct genus of lace bugs (Insecta: Heteroptera: Tingidae) occurring in latest early Eocene deposits of the Green River Formation, from the southern Piceance Basin of Northwestern Colorado, in North America. *Gyaclavator* can be placed within the Tingidae with certainty, perhaps it is sistergroup to Cantacaderinae. If it belongs to Cantacaderinae, it is the first fossil record of this group for North America. *Gyaclavator* has unique, conspicuous antennae bearing a specialized, highly dilated distiflagellomere, likely important for intra- or intersex reproductive competition and attraction. This character parallels similar antennae in leaf-footed bugs (Coreidae), and probably is associated with a behavioral convergence as well.

## Introduction

The Tingidae (lace bugs) belong to the infraorder Cimicomorpha and comprise more than 2100 described living species that are classified into three subfamilies, namely Cantacaderinae, Tinginae and Vianaidinae [1]. All tingids are herbivorous, including members of Vianaidinae, a small subfamily which members live in the nests of ants and likely feed on rootlets that penetrate the interstices of such habitats [2, 3]. As a group, lace bugs prefer woody plants and are host-specific piercers and suckers, being either monophagous on particular host species or oligophagous on species related at the generic level [4]. Atypical associations include species of the genus *Acalypta* Westwood, 1840 [5], which are among the few heteropterans that feed on mosses [6], and members of the genera *Copium* Thunberg, 1822 [7] and *Paracopium* Distant, 1902 [8], the only heteropterans that induce galls in their host plants [9].

The common name, lace bug, refers to the distinctive lace-like cuticular lattice on the hemelytra and on the pronotum of two of the three subgroups, the Cantacaderinae and the Tinginae; the third subgroup, Vianaidinae, has a rather smooth, partially punctate, body surface. Lace bugs are rare as fossils, but they have been described from both amber deposits and sedimentary rocks dating back to the late Mesozoic. Currently, there are 53 described fossil species of Tingidae. Two fossil species are affiliated with the Vianaidinae, but the position of *Vianathauma pericarti* Golub and Popov, 2003 [10], remains controversial [1]. Six fossil species belong to the Cantacaderinae, one of which is placed in the tribe Golmoniini Popov, 1989 [11]. The tribe Phatnomini includes 19 and the Tingini 18 fossil species, respectively, and seven species are assessed as incertae sedis. The position of *Burmacader multivenosus*, of latest Early Cretaceous age from Myanmar, remains unclear, as it shares a combination of characters representing different subfamilies of Tingidae [12]. A recent review of fossil tingid taxa is given by Wappler [13], supplemented by subsequent additions [10, 12, 14–28].

In an ongoing study of heteropterans from the southern Piceance Basin of the Green River Formation several male tingid specimens were discovered that exhibit an extremely enlarged antennal distiflagellomere. This feature is recorded for the first time for Tingidae, and a new genus and species are described. This account brings the number of named tingid species in fossil deposits of the U.S.A. to six, and is the first formally described tingid species from the Eocene Green River Formation. Fossil specimens with such remarkable preservation and novel anatomical structures considerably expand our knowledge of Cenozoic Tingidae, and thus refine our understanding of tingid evolution and behavior [29, 30]. Similarly enlarged antennal segments are known principally from members of the heteropteran infraorder Pentatomomorpha, particularly within the Coreidae, and the behavioral implications of this convergent evolutionary trait are discussed later in this paper.

## Materials and Methods

The tingid specimens were collected by Mr. David Kohls and Mr. Louis Přibyl during the mid-1990s as a result of providing an anthropogenically unbiased, randomly sampled collection of Green River material for the National Museum of Natural History (USNM), in Washington, D.C. Their excavations occurred in deposits of ancient Lake Uinta, in the southeastern component of this seventeen-million-year-long lake system. The particular collection containing the tingid fossil originated from the eastern depositional center of Lake Uinta, the Piceance Basin, which was largely separated from its western counterpart by the north-to-south trending Douglas Creek Arch [31]. No special permits were required for the described study, which complied with all relevant regulations.

**Fig 1:**
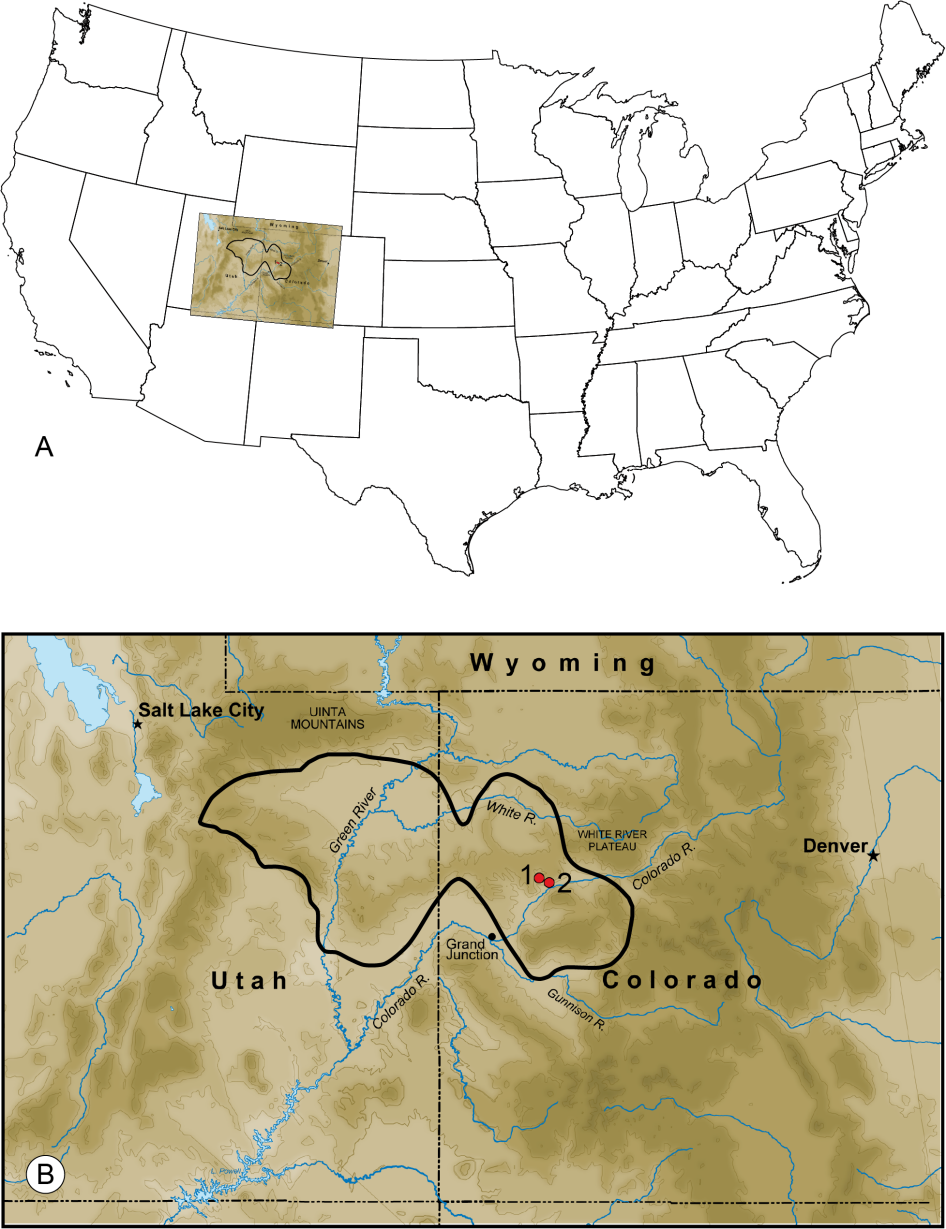
Locations of Green River Formation and fossil sites. A. Overview of continental USA (copyright 2004 Jared Benedict, licensed under the Creative Commons Attribution-ShareAlike License) with inset. B. Uinta-Piceance Basin of Green River Formation in Utah and Colorado. Red dots mark locations where fossils were found, 1 = Old Mountain Site, 2 = Denson Site; the black line outlines former Lake Uinta.

All specimens of the present taxon and associated metadata are deposited at the Department of Paleobiology, National Museum of Natural History, Washington, D.C., U.S.A. Investigated specimens were collected at the Green River Formation, Parachute Member, in Garfield County, Colorado, U.S.A., at Denson Site, USNM localities 42053 and 41679, and at Old Mountain Site, USNM locality 40189 (Fig 1).

**Fig 2:**
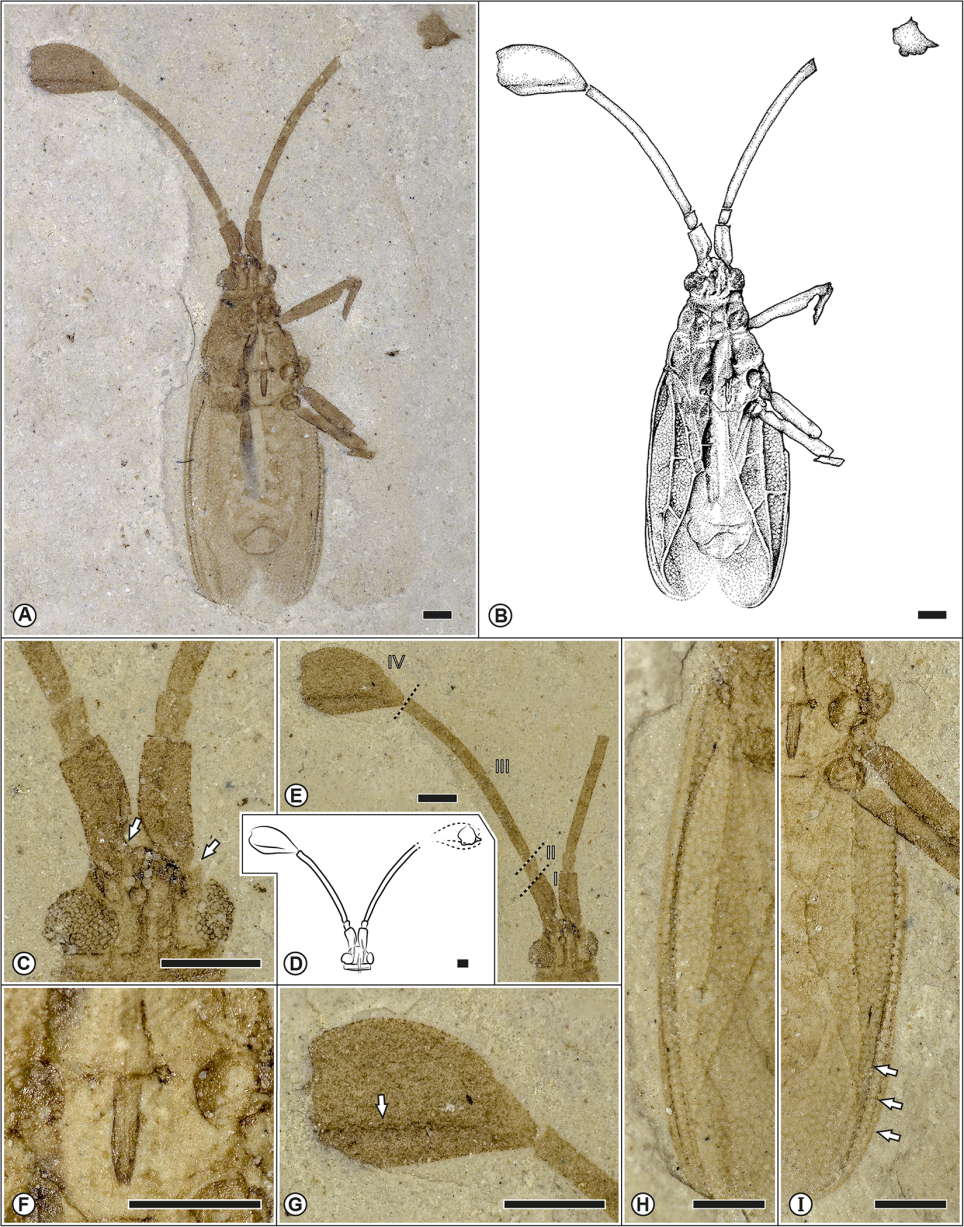
Different aspects on the morphology of *Gyaclavator kohlsi* gen. et sp. nov. A. Holotype. USNM 578190; photomicrograph of male in ventral view. B. Drawing of the holotype as preserved. C. Enlargement of ventral aspect of the head, arrows indicating the position of the bucculae (central) and the antennal process (right). D. Drawings of antennae from male specimens. E. Detail of the antennae. F. Labium. G. Enlargment of the petaliform last antennal segment, arrow indicate the position of the longitudinal ridge. H–I. Detail of the delicate and intricate network of divided areas in the hind wings, unlabelled arrows indicate the position of the stenocostal area. Scale bars represent 0.5 mm.

**Fig 3:**
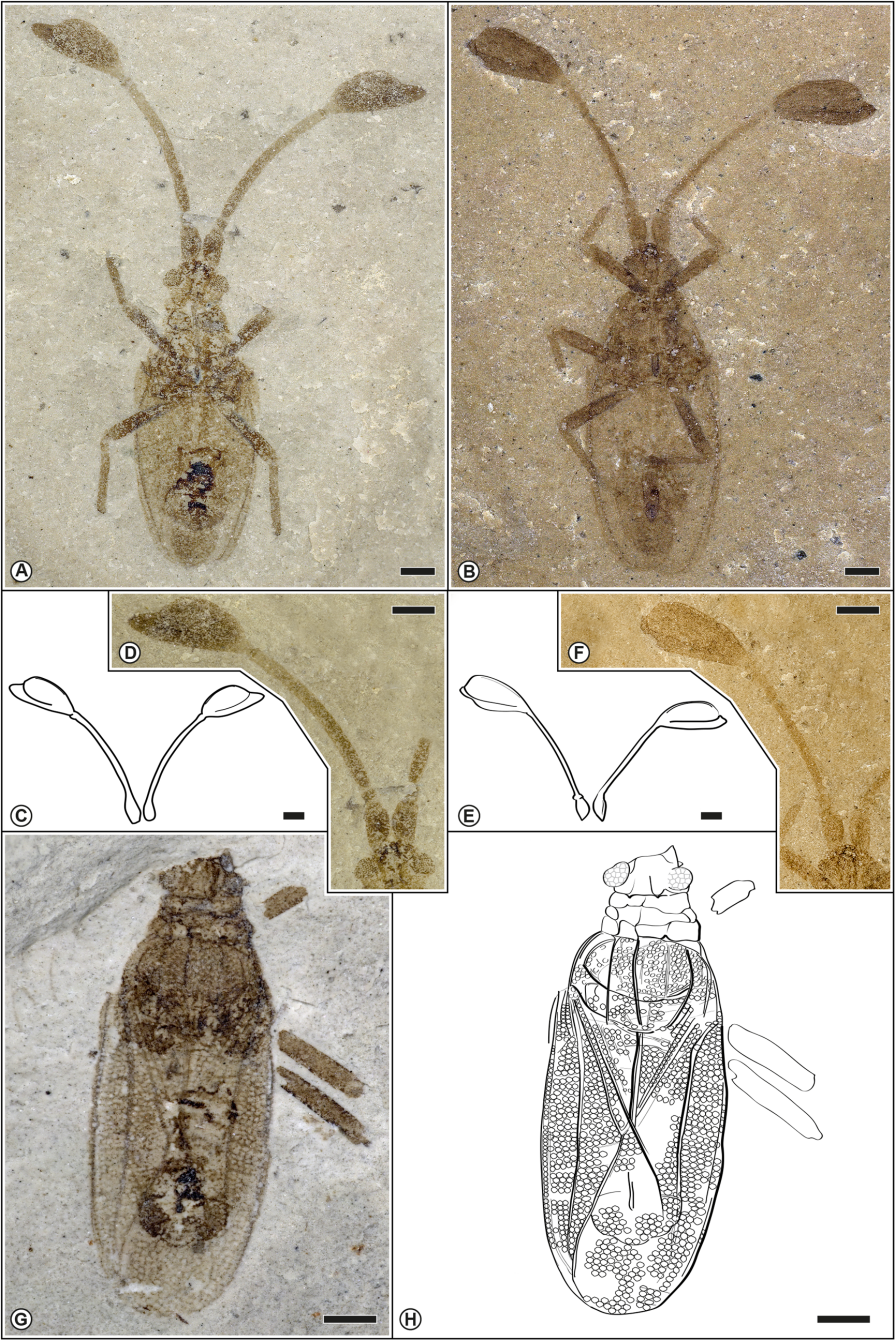
Morphological aspects of the paratypes of *Gyaclavator kohlsi*. A. Paratype. USNM 578171; male in ventral view. B. Paratype. USNM 572502; nearly complete male specimen in ventral view. C. USNM 578171; drawing of antennae. D. USNM 578171; detail of the antennae. E. USNM 572502; drawing of antennae. F. USNM 572502; detail of the antennae. G. Paratype. USNM 582493; photograph in dosal view. H. Paratype. USNM 582493; drawing of preserved insect. Scale bars represent 0.5 mm.

The four described tingid individuals were observed and digitized using a Keyence VHX-1000 microscope, and all relevant structures were measured from the digitized images. Drawings were produced directly from specimens using a camera lucida attachment on a Leica MZ16 microscope, except for Figs 2D, 3C, 3E and 3H, which were prepared as overlay drawings from photographs. Dashed lines indicate the edge of missing or otherwise obscured regions.

We follow the traditional systematic organization of Tingidae, that has been used, commonly accepted and discussed by Froeschner [32], Golub [33], Guilbert [34] and Schuh *et al*. [1] and the terminology by Froeschner [32].

### The Green River Formation

Much is known about the physical setting and geologic evolution of the Eocene Green River Formation, attributable largely to considerable economic interest in exploration of oil-shale layers within this spatiotemporally extensive complex of lake deposits in the western United States [31, 35]. However, the biota of the Green River Formation is less well understood [36], especially the insect fauna [37, 38]. The history of fossil insect studies of Green River strata has had a distinguished legacy extending to the pioneering work of S.H. Scudder in Wyoming during the 1890’s [39], and was soon followed by numerous contributions by T.D.A. Cockerell from the early 1900’s to the late 1920’s [40]. Summaries of the insect fauna have been made by Wilson [41], Grande [42, 43], and Graham [44]; however, these studies were regionally biased towards northern deposits of the Greater Green River Basin and Fossil Basin in southwestern Wyoming. More recently, several studies have focused on the insect fauna of the Piceance Basin in northwestern Colorado [45–52], and these reports provide a broad introduction toward understanding the insects along the southern margin of the basin, adjacent the Colorado River.

The Eocene Green River lake system developed primarily from exposure of erosion-resistant strata during multiple episodes of the Laramide Uplift, allowing widespread and prolonged deposition of organic-rich lacustrine mudstone [53] and associated finely laminated carbonate beds. These lithologies are exposed in various intermontane basins in northeastern Utah, southern Wyoming, and northwestern Colorado. The geological age of the major portion of the Green River Formation localities ranges from very early Eocene to mid middle Eocene on the basis of radiometric dating as well as mammalian biostratigraphy [31]. However, Lake Uinta was the longest lived of the Eocene lakes, spanning more than 17 million years, from late Paleocene to early late Eocene in age [31, 42]. The insect-yielding part of this sequence from the east-central Piceance Basin became substantially infilled with sediments, followed by freshwater flooding as Lake Uinta breached the Douglas Creek Arch [31].

There are three members of the Green River Formation within the Piceance Basin. The Parachute Creek Member represents the youngest member (ca. 47.8 Myr), characterized by considerable development of oil-shale sediments [31, 54]. To our knowledge, the Parachute Creek Member is the source of the insect and plant fossils for all the Piceance Basin localities, including those discussed herein.

The Green River paleoclimate has been determined by taxon-independent leaf physiognomy data using variables such as the size and shape of dicot leaves and by taxon-dependent analyses of nearest-living-relative for assessing the climatic associations of plant micro- and megafossils [55, 56]. Mean annual temperature is estimated as mid-mesothermal, ca. 16°C from leaf margin analysis, summarized in Wilf [56]. The presence of frost-intolerant taxa such as palms indicate mild winters [56].

### Phylogenetic Analysis

The relationships and the position of the newly described fossil were assessed on the basis of phylogenetic analyses. The terminal taxa and characters used in the analysis were taken from Guilbert [30] and Schuh *et al*. [1], with the addition of the newly described fossil. The resulting matrix contained 24 taxa and 50 characters from the adult morphology (Table 1; S1 Table); eleven of these were multistate characters. All characters were coded as unordered (Fitch parsimony) [57] and equally weighted. In all analyses *Xylastodoris* was used as an outgroup; it belongs to the miroid family Thaumastocoridae.

**Table 1:** Character matrix for extant and fossil Tingidae. Coding of characters see S1 Table.

Character	0	1	2	3	4	5	6	7	8	9	1	1	1	1	1	1	1	1	1	1	2	2	2	2	2	2	2	2	2	2	3	3	3	3	3	3	3	3	3	3	4	4	4	4	4	4	4	4	4	4
											0	1	2	3	4	5	6	7	8	9	0	1	2	3	4	5	6	7	8	9	0	1	2	3	4	5	6	7	8	9	0	1	2	3	4	5	6	7	8	9
*Xylastodoris*	-	0	1	2	-	-	0	0	0	0	0	1	0	0	0	0	0	-	-	0	0	2	1	0	2	0	0	0	0	0	0	1	-	0	3	0	0	0	0	0	0	0	0	-	-	0	0	0	1	-
*Onymocoris*	-	0	1	2	-	-	0	0	0	0	0	1	0	0	0	0	0	-	-	0	0	2	0	0	2	0	0	0	0	0	0	1	-	0	3	0	0	0	0	0	0	0	0	-	-	0	0	0	1	-
*Myiomma*	1	0	0	2	0	0	0	0	0	0	0	1	-	0	0	-	0	-	-	0	0	2	0	0	2	0	0	0	0	3	0	0	-	0	0	0	0	0	1	0	0	0	0	0	-	0	0	0	1	1
*Psallops*	1	1	0	2	0	0	0	0	0	0	0	1	-	0	0	-	0	-	-	0	0	2	0	0	2	0	0	0	0	3	0	0	-	0	0	0	0	0	1	0	0	0	0	0	-	0	0	0	1	1
*Plagiognathus*	1	1	0	2	0	0	0	0	0	0	0	1	-	0	0	-	0	-	-	0	0	2	0	0	2	0	0	0	0	3	0	0	-	0	0	0	0	0	1	0	0	0	0	0	-	0	0	0	1	1
*Anommatocoris bolivianus*	0	1	0	2	0	0	1	0	1	0	1	1	0	0	0	-	0	-	-	0	0	2	1	0	2	0	1	1	0	0	0	1	?	1	2	1	0	0	0	2	0	1	0	0	2	1	0	?	1	1
*Agramma*	1	1	0	0	0	0	0	1	1	0	1	0	0	1	0	0	0	0	0	0	1	1	-	0	0	0	0	1	1	2	1	-	0	1	0	0	0	0	0	0	0	1	0	1	1	1	0	1	1	0
*Corythucha*	1	1	0	0	0	0	0	1	1	0	1	0	0	1	0	0	1	0	0	0	1	1	-	0	0	0	0	1	1	2	1	-	0	1	0	0	0	0	0	0	0	1	0	1	1	1	0	1	1	0
*Tingis*	1	1	0	0	0	0	0	1	1	0	1	0	0	1	0	0	0	0	0	0	1	1	-	0	0	0	0	1	1	2	1	-	0	1	0	0	0	0	0	2	0	1	0	1	1	1	0	1	1	0
*Zetekella*	1	1	0	0	0	0	0	1	1	0	1	0	0	1	0	0	0	0	0	0	1	1	-	0	0	0	0	1	1	2	1	-	0	1	0	0	0	0	0	2	0	1	0	1	1	1	0	1	1	0
*Phatnoma*	0	1	0	0	1	0	0	1	1	0	1	0	0	0	0	0	0	1	0	0	1	0	-	0	0	0	0	0	0	2	1	-	0	1	0	0	0	0	0	2	0	0	0	1	1	1	0	1	1	0
*Allocader*	0	1	0	0	0	1	0	2	1	0	1	0	1	0	0	0	0	0	1	1	2	0	-	0	0	2	0	0	0	1	1	-	1	1	1	0	1	0	0	1	0	0	0	0	0	1	1	0	0	1
*Australocader*	0	1	0	1	0	0	0	2	1	0	1	0	1	0	0	1	0	0	0	1	2	0	-	0	0	2	0	0	0	1	1	-	1	1	1	0	1	0	0	1	1	0	0	0	0	1	1	0	0	1
*Cantacader*	0	1	0	0	0	0	0	2	1	0	1	0	0	1	0	0	0	0	0	1	2	1	-	0	0	2	0	1	0	1	1	-	1	1	1	0	1	0	0	1	0	0	0	0	0	1	1	0	0	1
*Carldrakeana*	0	1	0	0	0	0	0	2	1	0	1	0	0	0	0	0	0	0	0	1	2	0	-	0	1	1	0	0	0	1	1	-	1	1	1	0	1	0	0	1	0	0	1	0	0	1	1	0	0	1
*Ceratocader*	0	1	0	1	0	0	0	2	1	0	1	0	1	0	0	2	1	0	0	1	2	0	-	0	0	2	0	0	0	1	1	-	1	1	1	0	1	0	0	1	0	0	0	0	0	1	1	0	0	1
*Cyperobia*	0	1	0	0	0	0	0	2	1	0	1	0	0	0	1	0	0	0	0	1	2	0	-	0	1	1	1	0	0	1	1	-	1	1	1	0	1	0	0	1	0	0	0	0	0	1	1	0	0	1
*Nectacader*	0	1	0	0	0	1	1	2	1	0	1	0	1	0	1	0	0	0	0	1	2	0	-	0	0	2	0	0	0	1	1	-	1	1	1	0	1	0	0	1	0	0	0	0	0	1	1	0	0	1
*Pseudophatnoma*	0	1	0	0	0	0	0	2	1	0	1	0	0	1	0	0	0	1	0	1	2	1	-	0	0	2	0	0	0	1	1	-	1	1	1	0	1	1	0	1	0	0	0	0	0	1	1	0	0	1
*Stenocader*	0	1	0	0	0	0	0	2	1	0	1	0	0	0	1	0	0	0	0	1	2	0	-	1	1	1	0	0	0	1	1	-	1	1	1	0	1	0	0	1	0	0	0	0	0	1	1	0	0	1
*Teratocader*	0	1	0	0	0	1	0	2	1	1	1	0	0	1	0	0	0	1	0	1	2	1	-	0	0	2	0	0	0	1	1	-	1	1	1	0	1	0	0	1	0	0	0	0	0	1	1	0	0	1
*Caledoderus*	0	1	0	0	0	1	0	2	1	0	1	0	1	0	0	0	0	0	0	1	2	0	-	0	0	2	0	1	0	1	1	-	1	1	1	0	1	0	0	1	0	0	0	0	-	1	1	0	0	1
*Afghanoderus*	0	1	0	0	0	0	0	2	1	0	1	0	1	1	0	0	0	0	0	1	2	1	-	0	1	2	0	1	0	1	1	-	1	1	1	0	1	0	0	1	0	0	0	0	0	1	1	0	0	1
***Gyaclavator***	1	1	0	-	-	0	-	1	-	0	1	0	0	1	0	0	0	0	1	-	1	2	-	0	1	2	0	0	0	1	1	-	-	-	-	-	-	0	-	-	-	-	0	-	-	-	-	-	-	-

The parsimony analysis was conducted with the branch and bound algorithm of PAUP* 4.0b10 [58]. A strict consensus tree was calculated from the resulting most parsimonious trees. To test node stability a bootstrap analysis was done with the branch and bound algorithm and 1000 bootstrap replicates.

For a second methodologically independent approach not using parsimony as a criterion, we analysed the data with a probabilistic Bayesian algorithm using MrBayes 3.2.4 x64 [59, 60]. For this analysis, the datatype "standard" was used, as implemented for morphological data in MrBayes. Two analyses consisting of 2 million generations and two runs with four chains each were produced. One analysis was done with fixed equal rates for changes of all characters, and another analysis used gamma variable rates. The resulting topologies from both analyses were identical. WINCLADA [61] was used to evaluate the character distributions on the trees.

### Nomenclatural Acts

The electronic edition of this article conforms to the requirements of the amended International Code of Zoological Nomenclature, and hence the new names contained herein are available under that Code from the electronic edition of this article. This published work and the nomenclatural acts it contains have been registered in ZooBank, the online registration system for the ICZN. The ZooBank LSIDs (Life Science Identifiers) can be resolved and the associated information viewed through any standard web browser by appending the LSID to the prefix “http://zoobank.org/”. The LSID for this publication is: urn:lsid:zoobank.org:pub: A0F81E39-57EA-425E-B895-C0AED910339B. The electronic edition of this work was published in a journal with an ISSN, and has been archived and is available from the following digital repositories: PubMed Central, LOCKSS.

## Systematic Palaeontology

**Heteroptera Latreille, 1810** [62]

**Tingidae Laporte, 1833** [63]

***Gyaclavator* gen**. **nov**.

urn:lsid:zoobank.org:act:806CC8EA-5EF5-4A78-81F4-D3E118483925

### Type Species

*Gyaclavator kohlsi* sp. nov. (by monotypy).

### Diagnosis (based on male)

Macropterous. Body oblong, dorsal surface areolate. Head slightly produced in front of the compound eyes, bucculae not extended in front of the head. Antenna with segment I longer than II, II being shortest, III longest, IV strongly petaliform (autapomorphy of the genus). Pronotum with three longitudinal carinae of equal length; surface areolate; posterior margin rectilinear; comparatively short. Scutellum large, triangular. Hemelytra divided into stenocostal, costal, subcostal, discoidal and sutural areas by longitudinal elevated veins; surface covered with areolae, which are larger on costal area; transverse veins on subcostal and discoidal areas; clavus triangular.

### Comments

Although the overall preservation of the specimens is spectacular, many characters used in the study of Tingidae cannot be observed. However, sufficient detail can be extracted to provide a robust description and an assessment of the relationships of the new genus and species with phylogenetic analyses. The main autapomorphic feature of *Gyaclavator* gen. nov. is the specialized and unique antenna with a fourth antennal article that is distinctly petaliform and which justifies the erection of a new genus.

### Etymology

The new genus name is a combination from the Latin: *Gyas*, a name for a mythical giant, and *clavator*, meaning “club.” This binomial is a reference to the large size of the distiflagellomere. The gender is masculine.

### *Gyaclavator kohlsi* Wappler, Guilbert, Wedmann et Labandeira sp. Nov

(Figs 2 and 3)

urn:lsid:zoobank.org:act:D199D0AF-0A80-46FF-BAEB-1241B1079F0C

### Holotype

USNM 578190 (only one slab, no counterpart), deposited in the Department of Paleobiology, National Museum of Natural History, Washington, D.C., U.S.A. A well-preserved adult, ventro-dorsally compressed antennae well exposed (Fig 2); original field numbers shown in parentheses; David Kohls, collector.

### Paratypes

USNM 582493, (only one slab, no counterpart); Green River Formation, Parachute Member, uppermost lower Eocene (latest Ypresian); Old Mountain Site, USNM locality 40189, Garfield County, Colorado; USNM 572502 (part and counterpart), Denson Site, USNM locality 41679; USNM 578171 (only one slab, no counterpart), Denson Site, USNM locality 42053. David Kohls, collector.

### Etymology

The specific epithet derives from the surname of David Kohls, the collector of the type series, in recognition of his contribution to paleontology by the donation of this specimen to the National Museum of Natural History (USNM), Washington, D.C., U.S.A.

### Type Horizon and Age

Green River Formation, Parachute Creek Member. This is equivalent to the (latest) Ypresian Stage of the (latest) early Eocene.

### Type Locality

Denson Site, USNM locality 42053, Piceance Basin, Garfield County, Colorado, U.S.A.

### Diagnosis

As for the genus (see above).

### Description of holotype USNM 578190 (Fig 2)

Male. Macropterous. Body oblong, three times longer than wide; body length from head to tip of abdomen 4.6 mm, length from head to tip of hemelytra 5.5 mm; width of thorax 1.5 mm, width of abdomen 1.4 mm.

Head short with large eyes of nearly round outline, bucculae not extending forward at apex of clypeus, rostrum long, almost reaching abdomen; cephalic spines not evident, as specimen ventrally preserved; antennae as long as body length from head to abdomen, with four antennomeres; first segment stout, almost three times longer than second; second segment shortest, third segment longest, about five times longer than first; fourth segment stout, petaliform, much wider than others, about twice the length of the first, apparently with longitudinal ridge or divided in two adjacent structures, one longer, the other wider.

Pronotum longer than wide, collar narrow, with three apparently ridge-like carinae, narrow paranotum visible on right side, of small round areolae, narrow and/or reflexed onto the pronotum; sulcus present but metasternal laminae not seen; scutellum well developed, half the length of hemelytra. Rostrum almost reaching anterior border of abdomen. Femora rather stout, wider and shorter than tibiae, tarsi not seen.

Hemelytra of macropterous form; much longer than abdomen, slightly and typically widened at anterior part, round at posterior part; areolate, areolae generally small, round and of same size in all areas; divided in the usual areas. Stenocostal area narrow, with one row of areolae on posterior half; costal area narrow, wider than stenocostal area, widened in front and three to four areolae deep, with one row of areolae at the middle, with two rows of areolae directed backwards. (It is uncertain whether the hypocostal area obscures parts of the costal area; if so, the costal area is raised or slightly reflexed, unusual in Cantacaderinae.) Subcostal area much wider, five areolae wide at widest part, divided into several small areas by transverse veins; discoidal area as wide as subcostal area, five to six areolae wide at widest point, divided in four smaller areas by transverse veins; sutural area wide on posterior half; all main veins slightly raised.

### Paratype USNM 578171 (Fig 3A, 3C and 3D)

Male. Morphology very similar to holotype with characteristic antennae. Body lenght from head till end of abdomen 4.4 mm, length from head till tip of hemelytra 5.0 mm.

### Paratype USNM 572502 (Fig 3B, 3E and 3F)

Male. Morphology very similar to holotype with characteristic antennae. Body lenght from head till end of abdomen 4.5 mm, length from head till apex of hemelytra 5.4 mm. Rostrum long, almost reaching abdomen. Sutural area of macropterous hemelytra overlapping and projecting over apex of abdomen.

### Description of paratype USNM 582493 (Fig 3G and 3H)

Male. Morphology very similar to holotype with characteristic antennae. Anterior border of head not preserved, also no antennae preserved. Body length till end of abdomen ca. 4.3 mm, length head to hemelytra 5 mm. Areolae of the hemelytra equal in size, transparent. Anterior pronotal margin imperceptible. Pronotum with three parallel carinae. Stenocostal area uniserate. Costal area 0.3 mm wide, with four rows of rounded areolae. Subcostal area slightly wider than costal area, with six rows of cells in widest part. Discoidal area triangular in shape. Clavi not covered by pronotum.

## Results of Phylogenetic Analyses and Remarks

**Fig 4:**
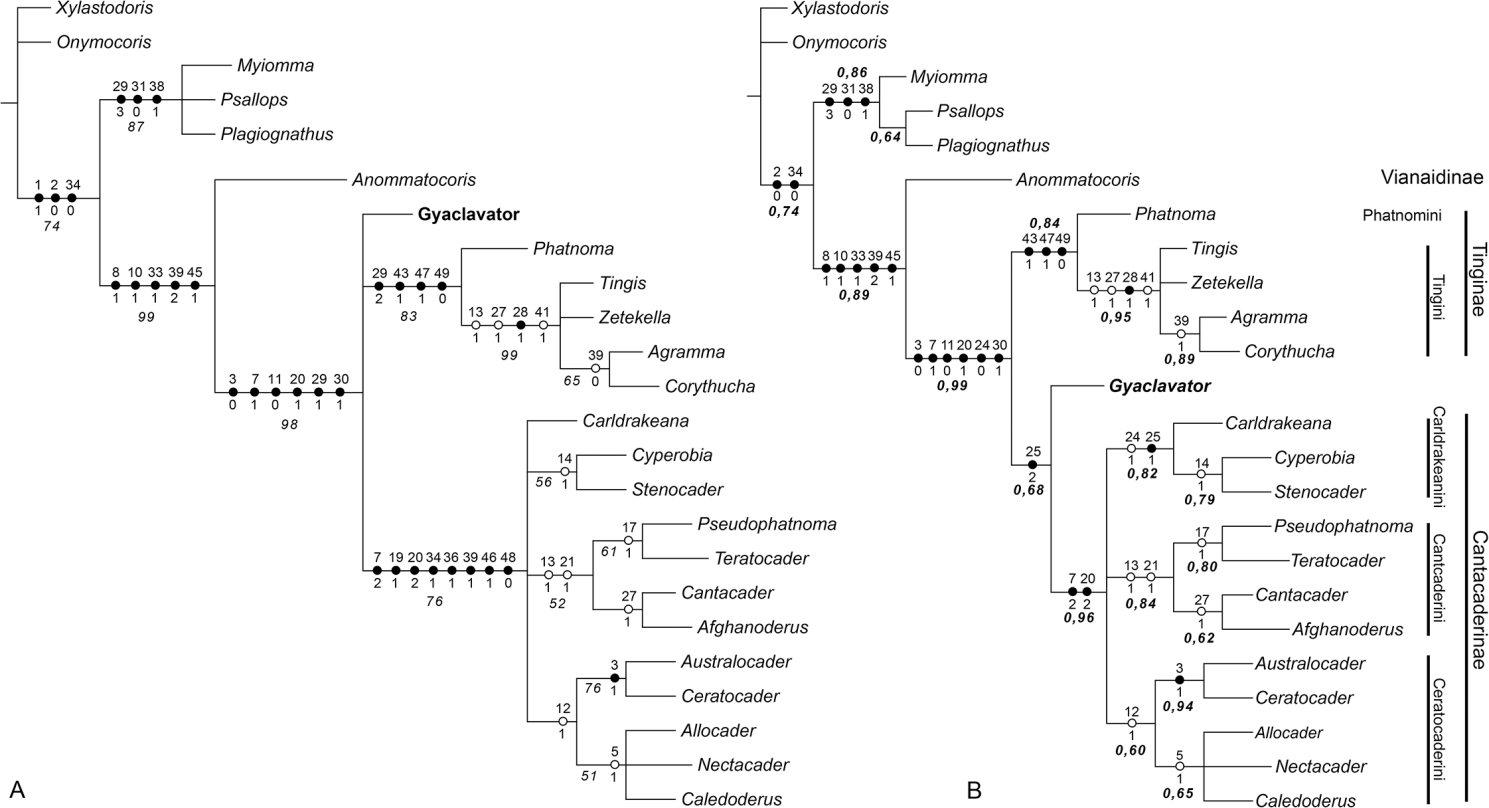
Phylogenetic relationships of *Gyaclavator*. Dots mark potential unambiguous autapomorphies. Solid black dots represent non-homoplasious, white dots represent homoplasious characters. Character numbers are placed above dots, characters states below dots. Autapomorphies of terminal taxa not shown. A. Strict consensus tree of 14 equally parsimonious trees from parsimony analysis. Numbers in italics are bootstrap values. B. Majority rule consensus tree from Bayesian analysis. Values in bold italics represent posterior probability of respective node.

The parsimony analysis with 50 morphological characters for 24 taxa resulted in 14 equally parsimonious trees with a length of 85 steps, each with a consistency index (CI) of 0.74, and a rescaled consistency index (RC) of 0.67. In all trees produced, *Gyaclavator* gen. nov. is included in the Tingidae. Within Tingidae, *Gyaclavator* n. gen. appears at varying positions in the 14 resulting, equally parsimonious trees. The strict consensus tree shows the genus in a trichotomy with Tinginae and Cantacaderinae (Fig 4A). In the results of the Bayesian analysis, *Gyaclavator* n. gen. appears also with a very high posterior probability within Tingidae, and with a posterior probability of 68%, it is the sistergroup to Cantacaderinae (Fig 4B).

Both cladograms in Fig 4 show that the clade *Gyaclavator* + Tinginae + Cantacaderinae is characterized by six unambiguous apomorphies (as indicated by the actual character number and states) (comp. S1 Table):

[3(0)] Spines on head straight.

[7(1)] The bucculae are in contact along the dorsal margin and do not surpass the apex of head. Bucculae are longer and are projected anteriorad of the head in the Cantacaderinae.

[11(0)] The second antennal article is considerably shorter than the third article.

[20(1)] Pronotal carinae are present.

[24(0)] Coastal area broad, more than 2 areolae medially (only in bayesian analysis).

[29(1)] Hemelytra with small irregular areolae (only in parsimony analysis).

[30(1)] The wing membrane disappears, while it is occasionally present in the Vianaidinae.

In the cladogram of the Bayesian analysis (Fig 4B) the sistergroup position of *Gyaclavator* to the extant Cantacaderinae is supported only by one potential synapomorphy [25(2)]: the ventrally and dorsally developed stenocostal area of the hemelytra. The monophyly of extant Cantacaderinae is supported by two characters, namely [7(2)], the bucculae projecting in front of head, and [20(2)], the five pronotal carinae present. Fig 4A shows that in the parsimony analysis, this clade is supported by many additional characters.

In view of these results, it seems possible, but it is not sure, that *Gyaclavator* was the sistergroup of the extant Cantacaderinae.

## Discussion

### Phylogenetic Aspects

For the fossil record of Tingidae, many fossil species have ambiguous phylogenetic relationships and exhibit a mosaic of characters typical for different taxa. For example *Archeopopovia* and *Tingicader* exhibit a combination of characters of the Phatnomini and Tingini. Other fossil taxa such as *Burmacader multivenosus* exhibit a combination of characters of the Cantacaderinae and Tinginae. A similar situation exists for *Gyaclavator kohlsi*. Unfortunately, diagnostic characters of the cephalic tubercles and the pregenital segments are not available for a better resolution of *Gyaclavator’s* taxonomic status, but *Gyaclavator* is distinguished from Tinginae + Cantacaderinae by the relatively large and triangular scutellum (Fig 3G), a character shared with members of the Vianaidinae. By contrast, the scutellum is greatly reduced in Tinginae + Cantacaderinae. The bucculae do not extend in front of the head (Fig 2C), which could argue for placement of *Gyaclavator* within Tinginae. Conversely, the ventral and dorsal presence of a uniseriate stenocostal area (Fig 2H–2I) and occurrence of transverse veins on the subcostal and discoidal areas argue for placement of *Gyaclavator* in the Cantacaderinae. The phylogenetic analyses of this complex mixture of characters favor slightly its close relationship with Cantacaderinae.

### Biogeographical Aspects

The Tingidae, particularly the Phatnomini, were very diverse in the Palearctic region during the middle Eocene. Fossils of Phatnomini are known from Northeastern Europe to Mongolia and from the Lower Cretaceous to the upper Miocene. The earliest fossil record belonging to the Phatnomini is *Sinaldocader ponomarenkoi* Golub and Popov, 2008 [20], from Baissa in Transbaikalia of Russia. The age of this specimen is mid Lower Cretaceous, likely Hauterivian to Barremian, ranging from ca. 125–135 Myr. Cantacaderinae fossils, or more precisely, Cantacaderini (because there are no fossil records yet for Carldrakeanini and Ceratocaderini), are more recent lineages and have been found from the European Eocene to the Miocene of northern Europe. Similarly, fossils of Tingini are relatively recent, occurring from the Eocene to Miocene, but are more biogeographically widespread, and are present from China via Europe to North America. Today, Tingini are worldwide distributed. Both the Cantacaderini and Phatnomini currently are distributed principally in tropical and subtropical regions of the southern hemisphere, while their fossils to date are mainly known from the Palearctic region. The third monophyletic group of Tingidae, the Vianaidinae, is represented by at least one fossil species in North America, from New Jersey amber of late Turonian age (ca. 93 Myr) [1, 64]. The Vianaidinae are basal to all other Tingidae but their extant distribution is restricted to South America. This could argue for a New World origin, buttressed by the fossil Vianaidinae from North America. The recent discovery of a fossil close to Vianaidinae in Burmese amber from the earliest Cenomanian at ca. 100 Myr [12] contradicts a New World origin for the clade, and instead suggests already a wide distribution during the Cretaceous. This scenario is also supported by the ancient species *Golmonia pater* Popov, 1989 [11], from the Lower Cretaceous of Mongolia (tribe Golmoniini), which has been considered as basal to Tingidae.

In conclusion, the origin and age of Tingidae are unclear. The oldest known fossil of Tingidae is a member of the Phatnomini, which is older than any fossils of the basal Vianaidinae. Known fossils of the Cantacaderinae are much younger than those of the Phatnomini. These data indicate that the Tingidae could be significantly older than the mid Early Cretaceous and more fossils are needed to shed light on their geographic origin.

If the new species *Gyaclavator kohlsi* belongs to the Candacaderinae, it would be the first fossil record of this group for the Nearctic region. Presently, no Cantacaderinae are known from the Nearctic region, but they occur in the Neotropics, and one fossil has been recorded from Dominican amber [14]. Consequently, their former occurrence in North America is not unexpected, although such a presence cannot contribute new insights to the questions concerning the biogeographic origin of the Tingidae.

### Behavioral Aspects

The dilated fourth antennal segment in *Gyaclavator kohlsi* n. sp. is probably a rare fossil example of a visual display structure that likely functioned for mate attraction or male-male-competition. This conspicuous feature previously was unknown for the Tingidae. Modified antennae also are found in the genera *Copium* and *Paracopium*, whereby the antennal segments can be strongly thickened; otherwise, these two genera differ minimally from Tinginae with only slightly enlarged antennae [e.g., 65] and they have nothing in common with the modifications found in *Gyaclavator*. The discovery of antennae with a communicative function in tingids is timely, as there has been considerable recent interest in the roles of sexual selection and reproductive competition in the evolution of insect reproductive behavior [66–69]. Adorned body parts such as antennae are mobile features that can be used as intraspecies semaphores for signalling with or without attractants, or with tactile stimulation [70]. Recently described flabellate antennae of a Lower Cretaceous hymenopteran indicate the antiquity of long-range female attractants [71]. In addition, it has been acknowledged that male reproductive success often is limited by access to females [72]; thus, sexual competition can select for secondary sexual characters in males. Consequently, visual displays such as in birds of paradise [73], peacocks [74] and claw waving displays of fiddler crabs [75], are conspicuous signalling structures that function in courtship and mate attraction, and probably are involved in male–male competition.

Speculation about which factors favor the dilation of antennal segments prominently include habitat effects, such as the availability of feeding resources, community composition or other environmental factors. It would be premature to attribute enlargement of the terminal antennal segment in *Gyaclavator kohlsi* to a particular factor, as sensillae are not preserved on the fossil specimens. However, examples are known of the dilation of particular antennal segments in coreid heteropteran adults, such as a the foliate or dialated third antennal article in the leaffooted bug *Chariesterus*, and the expanded second and third segments in *Chondrocera* [76]. In both examples, antennae are used for display and their conspicuousness through aerial waving may be partially associated with the presence of male neighbors and may function in male–male competition. Such displays also may act as a long-range female attractant (see S1 Movie). From these data, we infer that *G*. *kohlsi* represents an extreme specialization of antennal structure that is interpreted as convergent evolution of structural traits in unrelated lineages. Furthermore, these data show an unexpected morphological disparity in lace-bug antennal structure, and shed new light on mechanisms of sexual selection involving intra- and intersex reproductive competition during the early Eocene.

## Supporting Information

S1 Table List of characters and character states for the phylogenetic analysis. Associated data matrix given in Table 1.
(DOC)

S1 Movie Movie showing a leaf footed bug (Heteroptera: Coreidae) waving its antenna. Recorded by Sebastian Büsse in November 2011, at Reserva Privada de Patrimonio Natural, in San Sebastian, Bolivia (Reserva No. 01202). Published under a CC BY license, with permission from Sebastian Büsse, original copyright 2011.
(MP4)
