# Supplementary material for: Morphological and Behavioral Convergence in Extinct and Extant Bugs: The Systematics and Biology of a New Unusual Fossil Lace Bug from the Eocene
Source: PLoS One. 2015 Aug 12;10(8):e0133330. doi: 10.1371/journal.pone.0133330 (PMC4534043; doi:10.1371/journal.pone.0133330)
Supplement: S1 Table — Associated data matrix given in Table 1. (DOC) [file pone.0133330.s001.doc]

**S1 Table. List of characters and character states for the phylogenetic analysis.**

Associated data matrix given in Table 1.

1. Head distinctly projecting in front of eyes, surpassing antennal segment 1 (0); short, not surpassing antennal segment 1 (1);
2. Ocelli present (0); absent (1);
3. Mandibular plates not enlarged (0); enlarged, extending to apex of clypeus (1);
4. Spines on head straight (0); broadly upcurved (1); absent (2);
5. Clypeal spine absent (0); present (1);
6. Clypeus short, reaching at most to apex of jugo-frontal spines, if spines present (0); long, clearly surpassing apex of jugo-frontal spines (1);
7. Margins of bucculae without pilosity (0); with short, stiff setae (1);
8. Anterior length of bucculae short, not surpassing apex of head (0); bucculae in contact along dorsal margin anterior to clypeus (1); distinctly projecting in front of head, sometimes in contact (2);
9. Posterior length of bucculae short to moderately long, never reaching prosternum (0); elongate, extending from clypeus to prosternum (1);
10. Labium shorter, not reaching genital segment (0); longer, reaching genital segment (1);
11. Labial groove on thoracic sternum absent (0); present (1);
12. Antennal segment II distinctly shorter than antennal segment 3 (0); subequal to antennal segment III (1);
13. Pronotum not prolonged posteriorly along lateral carinae (0); prolonged posteriorly along lateral carinae (1);
14. Pronotum not prolonged posteriorly along median carina (0); prolonged posteriorly along median carina (1);
15. Lateral margins of pronotum rectilinear (0); bisinuate (1);
16. Lateral margins of paranota without teeth (0); with a single tooth (1); with 6 to 8 teeth (2);
17. Hood on anterior part of pronotum absent (0); present (1);
18. Paranotum unilobed (0); bilobed (1);
19. Paranota parallel to pronotal disc (0); strongly reflected upwards (1);
20. Lateral carinae on collar absent (0); present (1);
21. Number of pronotal carinae, none (0); 1 to 3 (1); 5 (2);
22. Scutellum with a distinct tubercle posteriorly (0); covered by pronotum (1); relatively large, triangular (2);
23. Costal vein extending to apex of corium (0); extending to apex of membrane (1);
24. Lateral margins of hemelytra normally developed (0); serrate (1);
25. Costal area broad, more than 2 areolae medially (0); narrow, none or one areola medially (1); absent (2);
26. Stenocostal area absent (0); developed only ventrally (1); development ventrally and dorsally (2);
27. Veins r+m and cu in basal part of hemelytron separate from one another (0); fused (1);
28. Discoidal area with transverse veins (0); without transverse veins (1);
29. Clavus normally developed (0); weakly developed and depressed below level of mesocorium (1);
30. Hemelytra (punctation) punctate (0); with small irregular areolae (1); with larger areolae (2); impunctate (3);
31. Membrane in macropterous form normally developed (0); rudimentary or absent (1);
32. Membrane with closed cells (0); without closed cells (1);
33. Medial vein of hind wing acutely connected with cu (0); perpendicular to cu (1);
34. Ostiole of metathoracic glands absent or posterior on metepisternum (0); anterior on metepisternum (1);
35. Scent gland peritreme developed as an apically closed loop or absent (0); crevice-like (1); T-shaped (2); absent (3);
36. Evaporative area of metathoracic glands absent to moderately developed, never covering entire metepisternum (0); covering entire metepisternum (1);
37. Trochanters not fused with femora (0); fused with femora (1);
38. Femur without apical spur (0); with apical spur (1);
39. Meso- and metafemoral trichobothria absent (0); 3 or more present on meso- and metafemora (1);
40. Parempodia well developed, setiform (0); greatly reduced (1); absent (2);
41. Carinae on abdominal sternum 2 absent (0); present (1);
42. Pregenital abdominal segments only II and III fused (0); II, III, IV, or more fused (1);
43. Tubercles on abdominal sternum 2 (Lis 2-1) absent (0); present (1);
44. Abdomen (lateral sclerites) with a simple set of lateral sclerites (0); with a double set of lateral sclerites (1);
45. Apodeme beween laterotergite VIII and gonocoxopodite I completely developed and functioning as a muscle connection (0); rudimentary absent (1);
46. Female abdominal sternum VII simple (0); with posterior medial projection (1);
47. Gonoplacs strongly sclerotized (0); membranous (1);
48. Pseudospermatheca absent (0); present (1);
49. Rudimentary spermatheca present (0); absent (1);
50. Vagina (genital chamber) small (0); spacious, sac-like (1).
